# PROXIMAL FEMUR FRACTURE IN OLDER ADULTS: CORRELATION BETWEEN SURGICAL TREATMENT TIME AND MORTALITY

**DOI:** 10.1590/1413-785220243201e283822

**Published:** 2025-04-07

**Authors:** BRUNA GRANIG VALENTE, ALINE CREMASCO ROCHA, HENRIQUE CHIARINI BATISTELLA, CRISTIANE TONOLI VELOZO DE ANDRADE, CARLOS AUGUSTO DE MATTOS, CINTIA KELLY BITTAR

**Affiliations:** 1 Pontifícia Universidade Católica de Campinas, Faculdade de Ciências Médicas, Campinas, SP, Brazil.

**Keywords:** Osteoporosis, Femur fractures, Hip fractures, Osteoporose, Fraturas do Fêmur, Fraturas do Quadril

## Abstract

**Introduction:**

Osteoporosis impacts public health because of its high morbidity and mortality in older adults and high costs to public funds.

**Objectives:**

Analysis of the epidemiological profile, temporal distribution, deaths, and period from low-impact proximal femoral neck fracture to management in older adults people treated at a Tertiary Hospital.

**Methods:**

Cross-sectional, descriptive and retrospective study that analyzed 133 medical records involving fractures of the proximal femoral neck due to low-energy trauma from 2017 to 2020. Statistical analysis using the chi-square test, Student’s t-test and Fisher’s exact test.

**Results:**

Of the 133 medical records, there was a predominance of females (p < 0.01) with 93 (69.92%). As for age, the average is 79.87±8.23, median 81 years and range from 61-99 years. The months of May, June, and August were dominant (p > 0.05), 15 (11.28%), 10 (7.52%), and 21 (15.79%), respectively. The most common spot (p < 0.001) was the transtrochanteric region 105 (78.95%). In four years, the number of deaths was: 5(20.83%) in 2017, 4(23.53%) in 2018, 8(33.33%) in 2019, 7(10.29%) in 2020,with no significant changes regarding sex (p > 0.05).

**Conclusion:**

Most data were consistent with the literature. However, two differ, the transtrochanteric fracture and mortality in 2020. The decrease in deaths in 2020 is mainly due to the new surgery protocol within 48 hours of the fracture. **Level Of Evidence IV, Case Series.**

## RESUMO

**Introdução:**

A osteoporose impacta a saúde pública pela alta morbimortalidade em idosos e pelos altos custos aos cofres públicos.

**Objetivos:**

Análise do perfil epidemiológico, distribuição temporal, óbitos e período da fratura colo de fêmur proximal em baixo impacto, até a conduta em idosos atendidos no Hospital Terciário.

**Métodos:**

Estudo transversal, descritivo e retrospectivo que analisou 133 prontuários envolvendo fraturas de colo de fêmur proximal por trauma de baixa energia de 2017 a 2020. Análise estatística pelo teste qui-quadrado, T Student e Exato de Fisher.

**Resultados:**

Dos 133 prontuários houve predomínio do sexo feminino (p < 0,01) com 93(69,92%). Quanto a idade, média é 79,87±8,23, mediana 81 anos e variação de 61-99 anos. Predominou-se (p > 0,05) o mês de maio, junho e agosto 15(11,28%), 10(7,52%) e 21(15,79%), respectivamente. O local mais comum (p < 0,001) foi a região transtrocanteriana 105(78,95%). Nos quatro anos os óbitos foram: 5(20,83%) em 2017, 4(23,53%) em 2018, 8(33,33%) em 2019, 7(10,29%) em 2020 e não havendo alterações significativas quanto ao sexo (p > 0,05).

**Conclusão:**

A maioria dos dados foram condizentes com a literatura. Todavia, dois se destoam, a fratura transtrocantérica e mortalidade em 2020. A queda de óbitos em 2020 deve-se principalmente ao novo protocolo de cirurgia em 48 horas da fratura. **Nível de evidência IV, Série de Casos.**

## INTRODUCTION

Osteoporosis is a systemic skeletal disease that reduces bone mass and deteriorates its microarchitecture. Consequently, bone fragility and susceptibility to fractures increase^1^. After 50 years, the probability of a man suffering a fracture due to osteoporosis is approximately 20%, but for women this number is higher, affecting around 50% of the population^2^. This pathology is very discrete, and can be asymptomatic until the first fracture occurs, and the diagnosis is made by analyzing the patient’s history, complaints of low-impact fractures, laboratory tests, imaging tests, and, especially, bone densitometry^3^, as low mineral density in the bone of the femoral neck is an important predictor^4^. 

Fractures of the lower limbs, most common of which are of the proximal femur, are the main cause of functional loss in the older adults^5^. The main cause in these patients is the fall from one’s own height and the treatment of most fractures is surgical, while conservative treatment is more indicated for cases of incomplete fractures or without displacement^6^. Besides previous comorbidities, prolonged time of immobility in bed and hospitalization tend to rapidly worsen the patient’s clinical conditions. In older patients, the risk of death increases when the period between fracture and surgery is longer than 48 hours. For each day waiting for surgery about 4% of death possibility of the older adult with hip fracture increases^7^.

In this scenario, the trend of significant expansion of the Brazilian older adults’ population by 2050 stands out^9^. The number of people over 60 years old in 1991 was 10.7 million people, whereas in 2011 this group expanded to 23.5 million^10^. Thus, there is an emerging need for planning and public policies to meet the demands of the older adults, since this group is increasingly affected and prone to suffer from this pathology that has a great power of incapacitation and lethality.

In an attempt to reduce postoperative complications and mortality in this population group, the hospital where this study was conducted implemented the “Surgical treatment of fractures in older patients” protocol, which consists of surgical treatment in patients aged 60 years or older, who present femoral fractures, especially proximal ones (femoral head, femoral neck, transtrochanteric, intertrochanteric, subtrochanteric), evidenced by radiography, tomography or magnetic resonance imaging. Studies have shown that early corrective surgery was associated with better functional outcomes and shorter hospital stays, including reducing mortality by up to 20% in the 12 months after the fracture in individuals over 60 years old^9^.

This study aimed to analyze the epidemiological profile, temporal distribution, deaths, and period between the occurrence of fracture in older patients with proximal femoral fractures, due to low-energy trauma, between 2017 and 2020 operated by the Orthopedics and Traumatology service of a Tertiary Hospital.

## MATERIALS AND METHODS

This is a descriptive, cross-sectional, retrospective, and comparative study conducted at the Tertiary Hospital, approved by the Research Ethics Committee (13390519.6.0000.5481). Patients under 60 years and with fractures from high-energy trauma were excluded. Sex, age, month, seasons, day of the week, period of the day, period between fracture and conduct, and deaths were evaluated.

Data collected from the medical records were arranged in an Excel spreadsheet and the pivot table tool was used to gather the results and facilitate statistical analysis. Quantitative variables were expressed as mean and standard deviation, and qualitative variables as frequencies and percentages. The statistical tests were performed with a significance level of α = 0.05 and, therefore, 95% confidence. The chi-square test was used to evaluate the distribution of cases by sex, age group, month, season of the year, day of the week, period of the day, and time between fracture and management and deaths. To compare sex and period before or after the protocol with mean age, Student’s t-test was applied. The verification of the existence of an association between the variables: fracture spot and mortality with sex. The mortality period before or after the implementation of the protocol was performed using Pearson’s chi-square test and Fisher’s Exact test.

## Results

**Figure 1:**
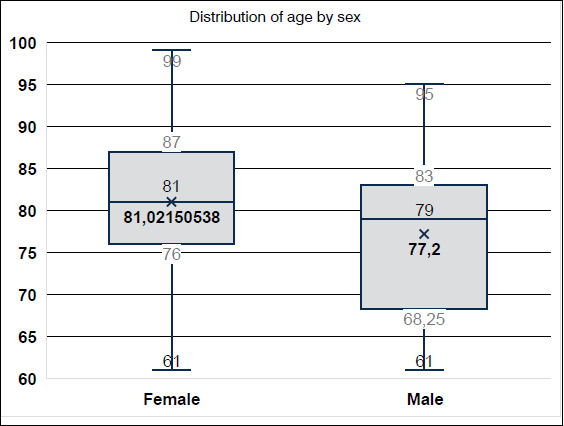
Patient distribution by sex.

**Source:** author’s own elaboration.

The study was composed of a sample of 133 medical records distributed over four years with 24 (18.05%) in 2017, 17 (12.78%) in 2018, 24 (18.05%) in 2019 and 68 (51.13%) in 2020, showing an increase of almost three times in the number of patients in 2020. **Table 1** and **Table 2** show that there was a relevant predominance (p < 0.01) of females (n = 93; 69.92%) over males (n = 40; 30.08%). Figure 1 shows that regarding age, the mean is 79.87±823, the median age is 81 years (ranging from 61 to 99 years), and the age group presents a significant bias (p < 0.001) for the prevalence of the 80-90 years interval. When sex was associated with the age of the patients, it was noticed that women were significantly older than men (p < 0.01), with a mean of 79.87±8.23 years.

**Table 1:** Epidemiological characteristics of older patients with femoral fractures

Characteristics of victims	2020		

	n	%	p-value*
**Sex**			p < 0.01
Male	40	30.08	
Female	93	69.92	
**Age**			p < 0.001
60-69 years	18	14	
70-79 years	41	31	
80-89 years	58	44	

(*) p-value by chi-square test using the homogeneity of the distributions each year as null hypothesis;

**Source:** author’s own elaboration.

**Table 2:** Mean age, fracture spot, and mortality

	Female (n = 93)			Male (n = 40)		
	n	%	p-value*	n	%	p-value*
Mean age	81.027.64		< 0.01	77.209		< 0.01
Type of fracture			< 0.001			> 0.05
Cervix	10	7.52		5	3.76	
Subtrochanteric	11	8.27		2	1.50	
Transtrochanteric	72	54.14		33	24.81	
Mortality						> 0.05
Deaths	15	11.28		9	6.77	
Non-deaths	78	58.65		31	23.31	

(*) p-value by chi-square test using the homogeneity of the distributions each year as null hypothesis

**Source:** author’s own elaboration.

**Figure 2:**
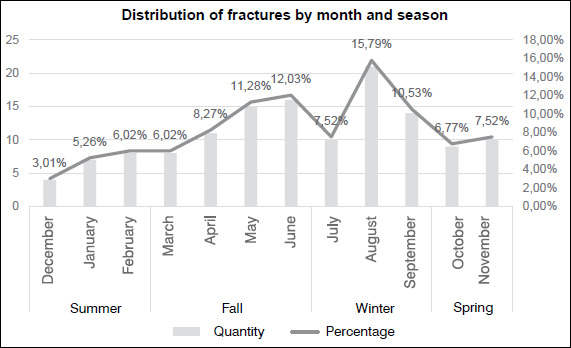
Distribution of fractures by month and season.

**Source:** author’s own elaboration.

**Figure 2** shows that there was a difference in the number of cases according to the month of the year (p < 0.05) being predominant in May (n = 15; 11.28%), June (n = 10; 7.52%), and August (n = 21; 15.79%). When analyzing the data assembled according to the seasons, the p-value < 0.01 helps to conclude that the cases are scattered in a heterogeneous way, being higher in winter (n = 48; 36.09%) and fall (n = 39; 29.32%).

P > 0.05 for the analysis of the distribution of fractures throughout the week indicates we cannot exclude the hypothesis that the cases occurred homogeneously, and it is not possible to affirm that any day of the week concentrated a greater number of cases. When analyzing the period of the day when the fractures occurred, data from the medical records that did not present this information were excluded from the calculation of the p-value, as named on **Table 1** as NI (n = 52; 39.10%), resulting in p < 0.01. P-value shows a statistically relevant bias for the heterogeneous distribution of cases, with a predominance of morning (n = 32; 24.06%) and afternoon (n = 21; 15.79%). 

**Table 2** shows a statistically significant difference (p < 0.001) regarding the fracture spots preferring unstable pertrochanteric femoral fractures (n = 105; 78.95%). There was no significant difference (p > 0.05) when correlating sex with the fracture spot.

In **Table 3** there was a significant reduction (p < 0.01) of the mean waiting time between the occurrence of the fracture and the surgical procedure increased from 6.69±8.85 days for the group evaluated before implementing the protocol (2017, 2018, and 2019) and 3.39±3.46 days for patients admitted after the protocol came into effect (2020). Of the total sample in the four years, 24 (18.05%) patients died within one year after the surgical intervention (between zero and eight months), which corresponds to 17 (26.15%) of the group analyzed before and seven (10.29%) of the group analyzed after the implementation of the surgery protocol within 48 hours after the occurrence of fracture in older adults. Note that there was a statistically significant decrease in mortality (p < 0.05) between both groups. There was no statistical correlation between sex and death (p > 0.05).

**Table 3:** Mean age, fracture spot, and mortality.

	Before the protocol	After the protocol	p-value
N	65	68	0.003*
MeanDP	6.698.85	3.393.46	
Median	5	2	
Maximum value	66	20	
Minimum value	0	0	
CI95%	2.19	0.83	
Mortality			< 0.05**
Non-mortality	48 (73.85%)	61 (89.71%)	
Death	17 (26.15%)	7 (10.29%)	

(*) T-test: two samples with unequal variances

(**) p-value by the chi-square test using the homogeneity of the distributions of each variable as a null hypothesis.

**Source:** author’s own elaboration.

## CONCLUSION

Osteoporosis is a global health problem due to the aging process of the population and has been characterized by WHO as the “Silent Epidemic of the Century”10. In Brazil, the number of people who have the disease reaches 10 million and it is estimated that treatment and care costs in the Unified Health System (SUS) with patients with osteoporosis and victims of falls and fractures will be around BRL 160 million in 2050^11^. This pathology has a high prevalence and predisposes the individual to falls and fractures that can lead to decreased functional capacity or even death^12^. Proximal femoral fractures have some special attention because they present a high risk for the older adults, it is estimated that mortality up to one year after the injury is around 20-30% and that only 15% fully recover the previous functional capacity, and 40% remain with severe disability^13^.

Most of our study samples agreed with results found in the literature. A statistically relevant predominance of fractures was observed in females (p < 0.01) in a 2.3:1 ratio, and mean age of 79.87±8.23 (between 61 and 99). Bittar et al. ^(^ ^14^ ^)^ found in this same service a predominance of women in a 4:1 ratio and a mean of 83.2 years (1999/2000). Rocha et al. ^(^ ^16^ found 3.3:1 and a mean of 78.5 years. Hungary et al. ^(^ ^15^ had a score of 2:1 and a mean of 78.2 years. Note that although similar studies show a higher incidence in the female population, there is no consensus on the ratio between the sexes and the mean age. Although osteoporosis affects both sexes equally, complications such as fractures tend to be more prevalent in women due to lower muscle strength, longer life expectancy, besides the climacteric and other comorbidities that are risk factors for this pathology. However, when a general analysis of fractures is performed, not focusing only on the proximal region of the femur, it is observed that men are the most affected, involving in most cases high-impact fractures, mainly related to traffic and work accidents^18^.

A correlation was observed, with a significant bias (p < 0.01), between sex and mean of age and it was observed that women’s mean (81.02±7.64) is higher than men’s (77.20±9). Bittar et al. ^(^ ^14^ ^)^ found 80.03 in women and 80.25 in men. Hungary et al. ^(^ ^12^ had 79.6 in women and 75.5 in men. Rocha et al. ^(^ ^16^ found 72.41 in women and 62.16 in men. Bagur et al. ^(^ ^17^ had 80 in women and 70 in men. In general, the studies show a higher prevalence of fractures in older women, when comparing our study’s results with the data in literature, the mean ages of both males and females are above the other studies quoted. The highest concentration of cases in the female population is due to the physiological loss of bone mass that begins after the age of 35 years and is more pronounced in females (1%/year) than in males (0.3%/year). Such reduction can be up to 10 times greater when a woman enters menopause^9^.

Regarding the temporal disposition of the 133 cases over the years, we have 24 (18.05%) in 2017, 17 (12.78%) in 2018, 24 (18.05%) in 2019, and 68 (51.13%) in 2020, showing an increase of almost three times in the number of patients in 2020. The significant increase in the number of cases is related to the fact that the hospital has become a reference institution for the care of traumas and other non-Covid pathologies in the city, as of April 2020^19^.

The distribution of cases per month is heterogeneous (p < 0.05) showing a higher concentration in May with 15 (11.28%), followed by June with 10 (7.52), and August with 21 (15.79%). When assembling the months by seasons, there was a predominance (p < 0.01) in winter 48 (36.09%) and fall 39 (29.32%), agreeing with various studies^13^ ^)-(^ ^15^, but there are studies that did not find this seasonal variation^13^. The reasons for the higher occurrence of winter fractures are uncertain, Chiu et al. ^(^ ^20^ try to explain this event with the hypothesis that the greater number of layers of clothing worn during the cold ends up compromising the mobility of the older adults and increasing the fractures incidence.

This study analyzed the occurrence of fractures throughout the week and, as well as the findings in literature, it did not show a relevant correlation (p > 0.05) to exclude the null hypothesis that the cases are arranged homogeneously, so it is not possible to affirm that any day of the week has concentrated a greater number of fractures^21^.

When evaluating the distribution by the period of the day, it showed significance (p < 0.01), with a higher frequency during the day, morning with 32 (24.06%) and afternoon with 21 (15.79%). However, this conclusion should be made with reservations due to the removal of the medical records that did not contain these data. The higher prevalence of fractures during the day is related to the performance of basic and instrumental activities of daily life. However, there are studies that obtained a higher prevalence at night, influenced by psychotropic medications^22^.

The fracture spot is associated with the anatomical region and with the aid of imaging tests. Thus, femoral neck fracture is related to injuries to the femoral neck, transtrochanteric is related to damage to the intertrochanteric line, and subtrochanteric injury occurs below the smaller trochanter^23^. There is no agreement among the studies on the ratio between the affected spots, but the literature presents the femoral neck as the region that suffers the most fractures. Ramalho et al. ^(^ ^24^ reported 49.3% of trochanteric fractures and 50.7% of femoral neck, whereas Bentler et al. ^(^ ^25^ ^)^ found 45% of trochanteric fractures and 55% of femoral necks. Our study showed results that differed, with 88.72% of the trochanteric fractures (78.95% transtrochanteric and 9.77% subtrochanteric) and 15% were of the femoral neck. However, such results agree with Bittar et al. ^(^ ^14^ ^)^ who performed the research at the same service (1999/2000) and obtained 80% of the trochanteric fractures (75% transtrochanteric and 5% subtrochanteric) and 20% of the femoral neck. 

The hospital implemented the protocol for the care of older adults with proximal femoral fractures, which consists of surgical intervention up to a maximum of 48 hours after their hospitalization. Thereby, a significant decrease (p < 0.01) of the mean time of surgical treatment can be seen, from 6.69±8.85 to 3.39±3.46 days after protocol implementation. Such decrease had a positive impact on the mortality analysis, with a significant reduction (p < 0.05), decreasing from 26.15% to 10.29% after the protocol. Our results agree with the literature, Siegmeth et al. ^(^ ^26^ ^)-(^ ^27^ who concluded an increase in mortality of patients over 60 years old when the surgical procedure is performed after 48 hours. Therefore, it showed a tendency to reduce the length of hospital stay and mortality by 6 months when surgery for the treatment of proximal femoral fractures in older adults is performed within 48 hours of hospital stay.

## CONCLUSION

Osteoporosis is considered a global health problem due to the aging process of the population. Most data were consistent with the literature. However, two are at odds, the transtrochanteric fracture and mortality in 2020. The main hypothesis that justifies the drop in deaths in 2020 was the implementation of the protocol “Surgical treatment of fracture in older patients”, which consists of surgical treatment in patients over 60 years of age within 48 hours after the fracture. The research emphasizes the importance of greater attention from hospital services in the care of older adults with fractures, as early treatment can modify and reduce morbidity and mortality rates, with reduced waiting times between fracture and surgery, and consequently reduce the length of hospital stay and the incidence of infections.
